# The DONALD study as a longitudinal sensor of nutritional developments: iodine and salt intake over more than 30 years in German children

**DOI:** 10.1007/s00394-022-02801-6

**Published:** 2022-01-18

**Authors:** Thomas Remer, Yifan Hua, Jonas Esche, Michael Thamm

**Affiliations:** 1 DONALD Study Center Dortmund, Department of Nutritional Epidemiology, Institute of Nutrition and Food Science, University of Bonn, Heinstück 11, 44225 Dortmund, Germany; 2 Department of Epidemiology and Health Monitoring, Robert Koch-Institute, Berlin, Germany

**Keywords:** 24-h urine, Iodine excretion, Iodine nutrition, Iodized salt, Longitudinal cohort, Sodium excretion

## Abstract

**Purpose:**

Mild-to-moderate iodine deficiency was present in large parts of Germany up to the beginning 1990s and improved from then on. Current epidemiological data on spot urine iodine measurements in German children strongly suggest the re-occurrence of an impaired iodine status. We thus examined whether this re-occurrence is identifiable in more detail, through iodine analyses of 24-h urine samples of a well-characterized cohort of German children in whom samples have been systematically collected from 1985 onward. As iodized salt is a major source for iodine supply, urinary sodium excretion was additionally studied.

**Methods:**

Daily iodine and sodium excretions were measured in 2600 24-h urine samples collected between 1985 and 2018 by 677 healthy children aged 6–12 years (participants of the DONALD study). These data were compared with 24-h iodine and sodium excretion estimates obtained from spot urine samples collected in the representative German Health Interview and Examination Surveys for Children and Adolescents KiGGS-baseline (2003–2006) and KiGGS-wave-2 (2014–2017).

**Results:**

Between 1985 and1992, DONALD participants started with a median daily iodine excretion level of 40.1 µg/d. Then, during 1993–2003, iodine excretions mounted up to an approximate plateau (~ 84.8 µg/d). This plateau lasted until 2012. Thereafter, iodine concentrations started to decrease again resulting in a median iodine excretion of only 58.9 µg/d in 2018. Sodium excretion, however, had increased. The marked decrease in iodine status along with an abundant sodium excretion corresponded closely with nationwide KiGGS data.

**Conclusions:**

As exemplified for the clearly worsening iodine status in German children, longitudinal cohort studies collecting detailed biomarker-based prospective data have the potential to reliably capture health-relevant nutritional changes and trends, applicable on a more comprehensive and even representative population level.

**Supplementary Information:**

The online version contains supplementary material available at 10.1007/s00394-022-02801-6.

## Introduction

Germany is an iodine-deficient region [1] and goiter prevalence was above 10% up to the beginning 1990s in several German regions [2]. While in the 1980s adult males’ and females’ median daily iodine excretion was found to be only 64 and 52 µg/d, respectively [3], iodine nutrition considerably improved during the following decade and urinary excretion, on average, exceeded 100 µg/d in various groups of adults [3]. Also analyses in 24-h urine samples in school children, yielded a corresponding improvement from somewhat more than 60 µg/d/1.73 m^2^ to around 100 µg/d/1.73 m^2^ within an around 10 year period after the mid-1980s (median values each corrected for adult body surface area) [4].

Several years later the Robert Koch-Institute accomplished a nationwide representative survey in German children during the years 2003–2006 (KiGGS baseline) followed by a second one KIGGS wave 2 (KiGGS-2) around 11 years later (2014–2017). These surveys, done with spot urine collections, revealed a further improvement in iodine supply in the period 2003–2006 [5] and a decrease again in 2014–2017 [6].

We thus examined whether these cross-sectionally determined long-term ups and downs of iodine status with a recent re-occurrence of iodine deficiency [6] are specifically identifiable in more detail by iodine analyses in 24-h urine samples in a well-characterized cohort of German children in which samples have been longitudinally collected from 1985 onward. As along with milk and milk products, iodized salt, particularly contained in processed foods, is the most important source for iodine supply in Germany [7, 8], we additionally studied whether some kind of long-term reduction in salt intake—assessed via 24-h urinary sodium excretion measurements—might have contributed to the recent impairment in iodine status.

## Materials and methods

### Study populations and urine measurements

Analyses were done in three population samples: the DONALD (Dortmund Nutritional and Anthropometric Longitudinally Designed) Study and two nationwide studies termed German Health Interview and Examination Survey for Children and Adolescents (KiGGS), i.e., KiGGS-baseline and KiGGS-2.

#### DONALD

Data collection in the DONALD open cohort study started in 1985 with the aim to prospectively gather information on diet, metabolism, growth and development from infancy to adulthood in healthy individuals [9].

In short, after parents agreed to participate, 3-d weighed dietary records as well as anthropometric and anamnestic data are collected from the first year of life onward in children medically confirmed to be healthy. Around 35–40 infants are newly recruited every year. At an age of 3–4-year-old children are asked whether they are willing to provide—once yearly around their birthday—a 24-h urine collection [9, 10]. Participation rate in urine collection of the 3-year-olds is about 15% and of the 6-year-olds almost around 50%. The DONALD study protocol was approved by the Ethics Committee of the University of Bonn, Germany, and all assessments were performed with parental and grown-up children’s consent.

For the present investigation, all participants of the DONALD study aged 6–12 years who had collected at least one 24-h urine sample between 1985 and the end of 2018 were included. Up to the early nineties not only babies but also preschool and school children had been recruited so that already from 1985 onward 24-h urines were available for children of 6 years and older.

Urine collections were performed at home using 1-L plastic containers (Extran-cleaned [Extran, MA03; Merck, Darmstadt, Germany], preservative-free) and were stored at ≤ − 20 °C until being thawed for analyses. Children and/or their parents were carefully instructed in the collection procedure for the 24-h urine sample and received additional written guidance. For determining completeness of the urine collection, children recorded times of each micturition (the younger ones with the help of one parent). A dietitian explicitly asked about each child’s compliance [11]. Samples containing one or more micturitions with unknown losses were not included in the study. Data on the actual total collection interval were used to individually correct measured 24-h urine volumes. To further minimize errors from urine collection, samples with daily creatinine excretion < 0.1 mmol/kg body weight were excluded from analysis. Overall compliance in collecting acceptable 24-h urine samples was around 80% for the 6–12-year-old children. Further details on urine collection and anthropometrics are given elsewhere [12, 13].

In the DONALD Lab urinary iodine concentration was measured using a modified Sandell–Kolthoff method after acidic wet-ashing of the samples with perchloric acid [14]. This method and the Sandell–Kolthoff procedure used in the central epidemiology laboratory of the Robert Koch Institute (RKI) were both validated against the gold standard analytics for iodine measurements, i.e., the inductively coupled plasma mass spectrometry (ICP-MS) yielding close agreement with the ICP-MS in the most relevant concentration range 50–100 µg/L for both laboratories [14]. Imprecision and variation was somewhat higher in the high-concentration domain for the RKI laboratory with intraclass correlations in comparison to ICP-MS of 0.91 vs. 0.98 (DONALD Lab). Analyzing different levels of the same urine-reference materials yielded recoveries of 100–108% and 96–103% for the RKI and the DONALD Lab, respectively. Both Labs have participated in validation checks of the EQUIP program [14].

24-h Na excretion was analysed by flame atomic absorption spectrometry with a Perkin Elmer 1100 Spectrometer (Perkin Elmer, Überlingen, Germany). Creatinine concentration was quantified in all samples by the kinetic Jaffe method with the use of a creatinine analyser (Beckman-2; Beckman Instruments, Inc., Fullerton, CA, USA).

#### KiGGS-baseline and KiGGS-2

The German health monitoring system conducted by the Robert Koch Institute has accomplished up to now two representative cross-sectional examinations in children: KiGGS-baseline in the years 2003–2006 and KiGGS-2 eleven years later in period 2014–2017. In both surveys more than 15,000 children and adolescents covering the age range from 0 to 17 years were selected randomly from official population registries of cities and municipalities representative for Germany.

Self-administered questionnaires on physical and mental health, health-related behavior (such as food intake and physical activity) and further prevention-relevant, social, and environmental factors were completed by the participants or their parents or legal guardians in KiGGS-baseline and KiGGS-2. In the former, spot urine samples were collected from 14,134 and in the later from 3,364 children and adolescents.

From both surveys, all 6–12-year-old children were selected for whom spot urine collections were available with completed measurements of urinary iodine and creatinine concentrations [15]. Measurements were performed in the central epidemiology laboratory of the Robert Koch Institute: (i) urinary iodine determined after acidic ashing with ammonium persulfate by the Sandell–Kolthoff method on an analyzer system (Cobas Mira Plus; Roche, Basel, Switzerland), (ii) urinary creatinine by the colorimetric picrate method on the Architect platform CI 8200, Abbott, USA, and (iii) urinary sodium by an ion-sensitive electrode (ISE; indirect method), also on the Architect CI 8200 platform. Sodium measurements were only performed in KiGGS-2. Eventually, the final numbers of samples of 6–12-year-old schoolchildren from KiGGS baseline and KiGGS-2 included in the present study were 7211 and 1586, respectively.

KiGGS-baseline and KiGGS-2 were conducted in accordance with the Declaration of Helsinki. Written informed consent was obtained from parents and all participants aged 14 years and older before data collection. The protocol was approved by the Federal Commissioner for Data Protection and Freedom of Information and by the ethics committee of the Hannover Medical School (Number 2275–2014). Further details on design and methodology of the KiGGS surveys have been described in more depth elsewhere [16, 17].

### Urinary data

While in the DONALD study 24-h urine samples are collected, only spot urine collections have been done in both KiGGS surveys. To enable comparability between the studies, 24-h urinary iodine as well as 24-h urinary sodium output was estimated from spot iodine, sodium, and creatinine measurements referring to age and sex specific 24-h urinary creatinine reference values as described in detail repeatedly [7, 12, 18]. Salt intake (g/d) was then calculated from 24-h sodium excretion through multiplication by the factor 2.5.

### Statistical analysis

Statistical analyses were performed using SAS (version 9.2, SAS Institute, 107 Cary, NC, USA) with *p* values < 0.05 considered significant. Linear mixed-effects regression models (PROC MIXED) were used to analyze time trends in iodine and sodium excretion, taking into account the dependency between repeated measurements within the same child. A random statement defining random effects was included to allow for variation in initial iodine excretion levels between the individuals. According to the Akaike’s Information Criterion, an unstructured type of covariance was used as the best fit option in the random SAS commands specifying the covariance structure. PROC MIXED models were tested for linearity, heteroskedasticity, multicollinearity and normality of residuals. If residuals were not normal distributed, outcomes were log10-transformed. This was the case for the outcome 24-h urinary iodine excretion in two of 4 pre-specified observation periods, i.e., periods 1985–1992 and 2012–2018.

PROC MIXED analyses for iodine were done for each of the pre-specified periods 1985–1992, 1993–2003, 2004–2011, and 2012–2018 separately to test either within-period changes or the presence of a plateau. With an analysis over the whole observation span (1985–2018) the overall time trend for 24-h sodium excretion was examined. Apart from the predictor variable time (years) the covariates sex, age, and creatinine were allowed for in the PROC MIXED models. Regarding the outcome 24-h urinary iodine excretion, the respective per-day urine volume was additionally adjusted [19].

Comparisons of iodine levels between the four specified periods were done by Kruskal–Wallis tests followed by Wilcoxon rank sum tests with each child included only once in the analysis. Of those with more than one urine collection, PROC SURVEYSELECT was used to randomly select one urine sample per child and assign selected samples in an as far as possible balanced fashion to each time period. This allows use of the same test for comparisons between time periods as for comparison of KiGGS baseline vs. KiGGS-2 (Wilcoxon rank sum test).

Estimation of the proportion of iodized salt intake to total salt intake was done by calculation of the median amount of urinary iodine stemming from total salt intake (i.e., sodium excretion) according to Haldimann et al. [20]. In short, using linear regressions, predicted values for iodine intake (24-h iodine excretion + 10% for non-renal iodine loss) were calculated with salt intake (24-h sodium excretion × 2.5) as the predictor after adjusting for age, BMI, and sex. The proportion of iodine from salt was then deduced from a subsequent regression of the obtained predicted values of iodine intake on salt intake. For more details, see Esche et al. [7].

## Results

**Fig. 1:**
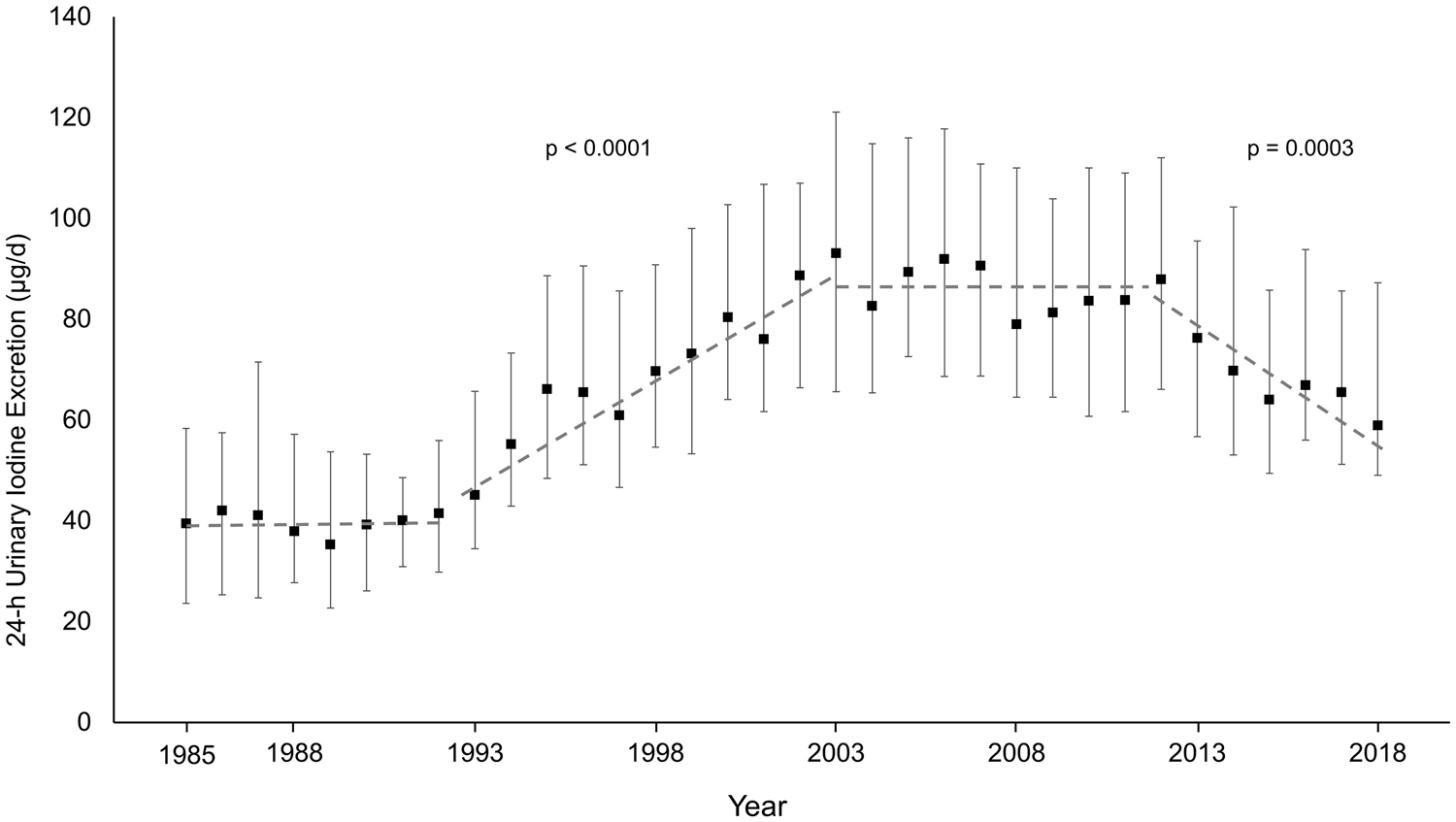
24-h urinary iodine excretion of DONALD participants aged 6–12 years from 1985 to 2018 (*n* = 959 children, 4028 urine samples). Data shown as median and interquartile range; significant time trends during periods 1993–2003 and 2012–2018, not significant during 2004–2011 and 1985–1992. Kruskal–Wallis test over all predefined observation periods of the total time span of 34 years (cross-sectional, balanced design; *p* < 0.0001) followed by Wilcoxon rank sum tests for comparisons of period 1 vs. 2 (1985–1992 vs. 1993–2003; *p* < 0.0001), period 2 vs. 3 (1993–2003 vs. 2004–2012; *p* < 0.0001), and period 3 vs. 4 (2004–2012 vs. 2013–2018; *p* < 0.0001)

**Fig. 2:**
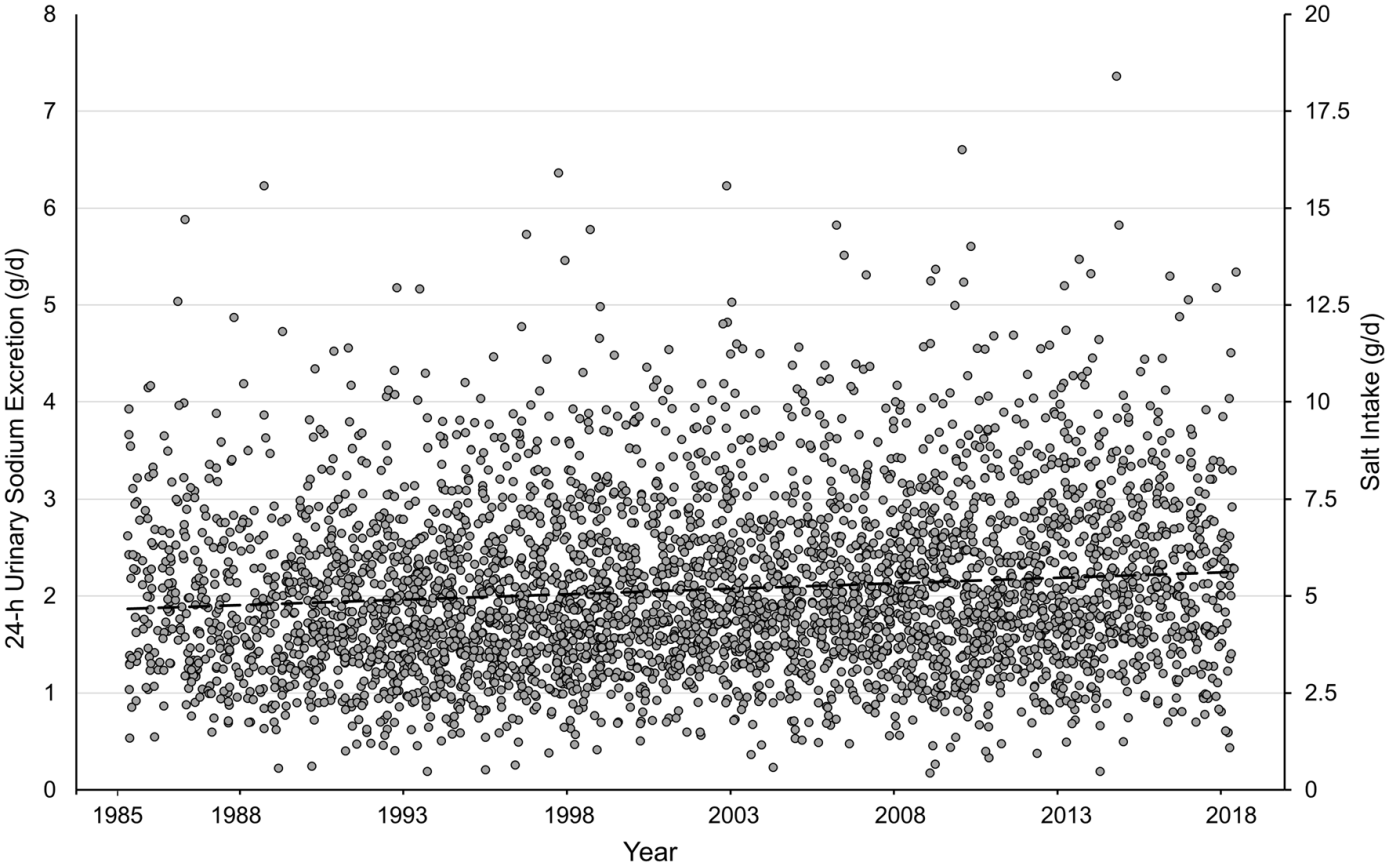
24-h urinary sodium excretion and excretion-derived salt intake of 6–12-year-old DONALD children over 34 years till 2018 (*n* = 959 children, 4028 urine samples). Salt intake (g/d) was calculated from 24-h sodium excretion through multiplication by the factor 2.5. Time trend for whole period 1985–2018: *p* < 0.0001, β value 0.022 (0.015, 0.029). Analyses were performed by linear mixed-effects regression models (PROC MIXED). Statistical variance components of the mixed-models are shown in Supplemental Table 1

24-h urinary iodine excretion of 6–12-year-old schoolchildren of the DONALD cohort exhibited a considerable alteration during the recent > 30 years. Iodine excretion was very low between 1985 and 1992 and thereafter increased markedly with a doubling of daily excretion rates from ca. 40 to > 80 µg/d (median level: 84.8 µg/d in 1993–2003) (Fig. 1). After almost 1 decade of scattering around this 85 µg/d level, a return decrease occurred with the lowest median excretion rate of 58.9 µg/d in 2018 (Fig. 1). Corresponding sodium excretion and the accordingly derived salt intake, however, steadily increased during these more than 30 years (Fig. 2).

**Fig. 3:**
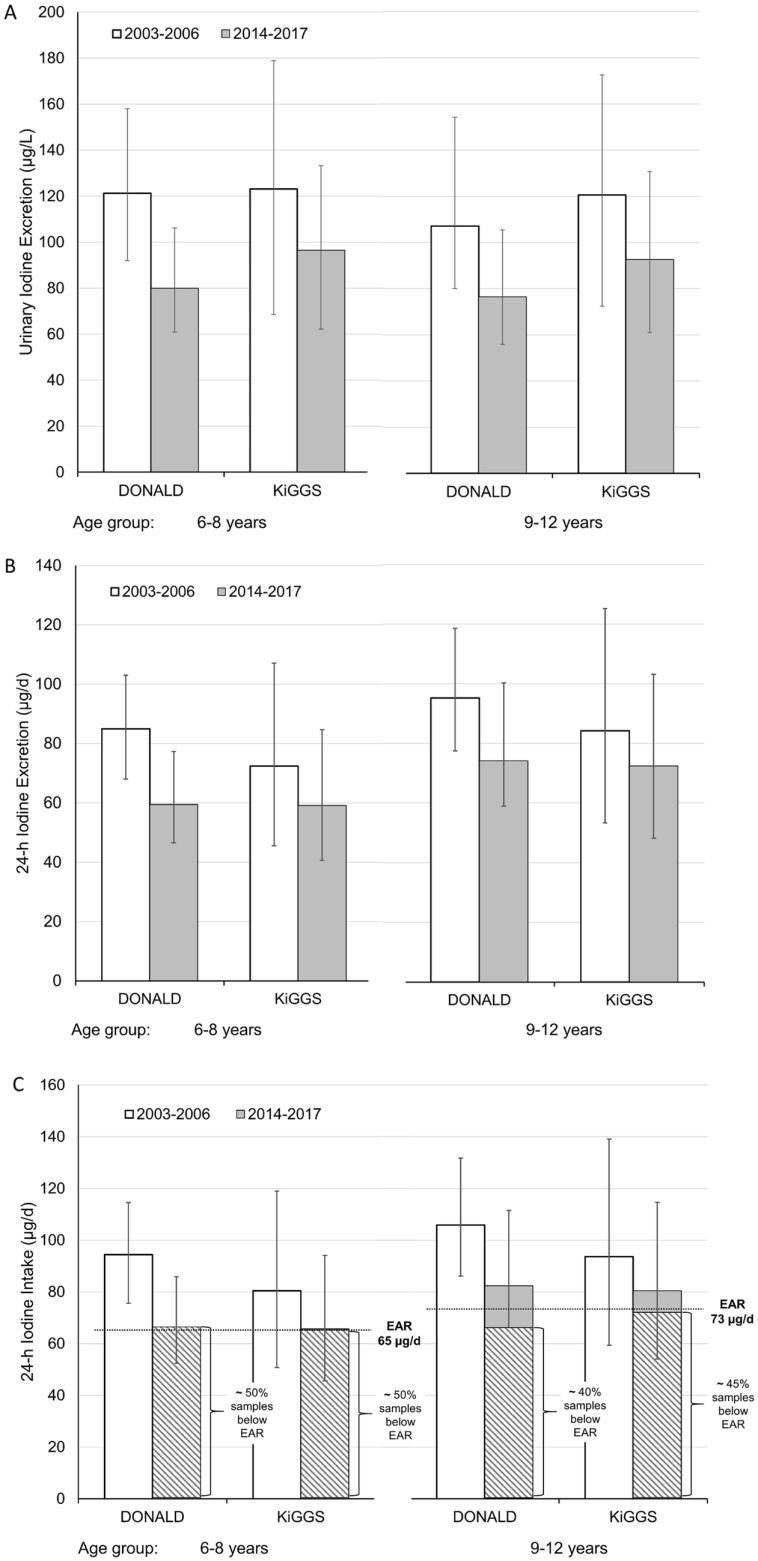
Reduction in iodine status of German schoolchildren within about 1 decade: comparison between changes in DONALD study and changes in nationwide KiGGS study waves. **A** iodine concentrations; **B** 24-h iodine excretion rates (those for the KiGGS children were estimated from spot urine data); **C** estimated daily iodine intake (assuming fecal iodine losses of 10%) showing that about 50% of the urine samples of the 6–8-year-old children and ca. 45% of the 9–12-year-olds of KiGGS-2 translate into daily iodine intakes below the respective estimated average requirement (EAR). Iodine excretions of DONALD children with more than one urine collection within a respective age group were averaged before statistical analysis. All changes in both study types were highly significant (*p* < 0.0001, Wilcoxon rank sum tests)

The nationwide surveys KiGGS-baseline and KiGGS-2 conducted in Germany in 2003–2006 and 2014–2017, respectively, revealed a clear significant decrease of iodine status in children as depicted in Fig. 3B for the 6–8-year and the 9–12-year-old children. Calculated 24-h iodine excretion in the former age group fell from median 72.4 µg/d to median 59.1 µg/d and in the latter from 84.2 µg/d to 72.4 µg/d. A more marked decrease in iodine excretion was discernible in DONALD children who started from a somewhat higher initial level. In period 2 (2014–2017) median excretion levels eventually were almost identical in KiGGS (59.1 and 72.4 µg/d) and DONALD (59.5 and 74.1 µg/d) (Fig. 3B).

These excretions amount to median daily iodine intake levels of around 66 µg/d and 81 µg/d for both studies KiGGS-2 and DONALD if average fecal iodine losses of 10% are assumed. Accordingly, representative spot urine measurements as well as specific cohort data on 24-h urine collections hint at a situation in Germany where about 50% (6–8 years old) or 45% (9–12 years old) of school children were at risk of iodine deficiency, i.e., their estimated iodine intakes are below the estimated average requirements (EAR) (Fig. 3C).

**Fig. 4:**
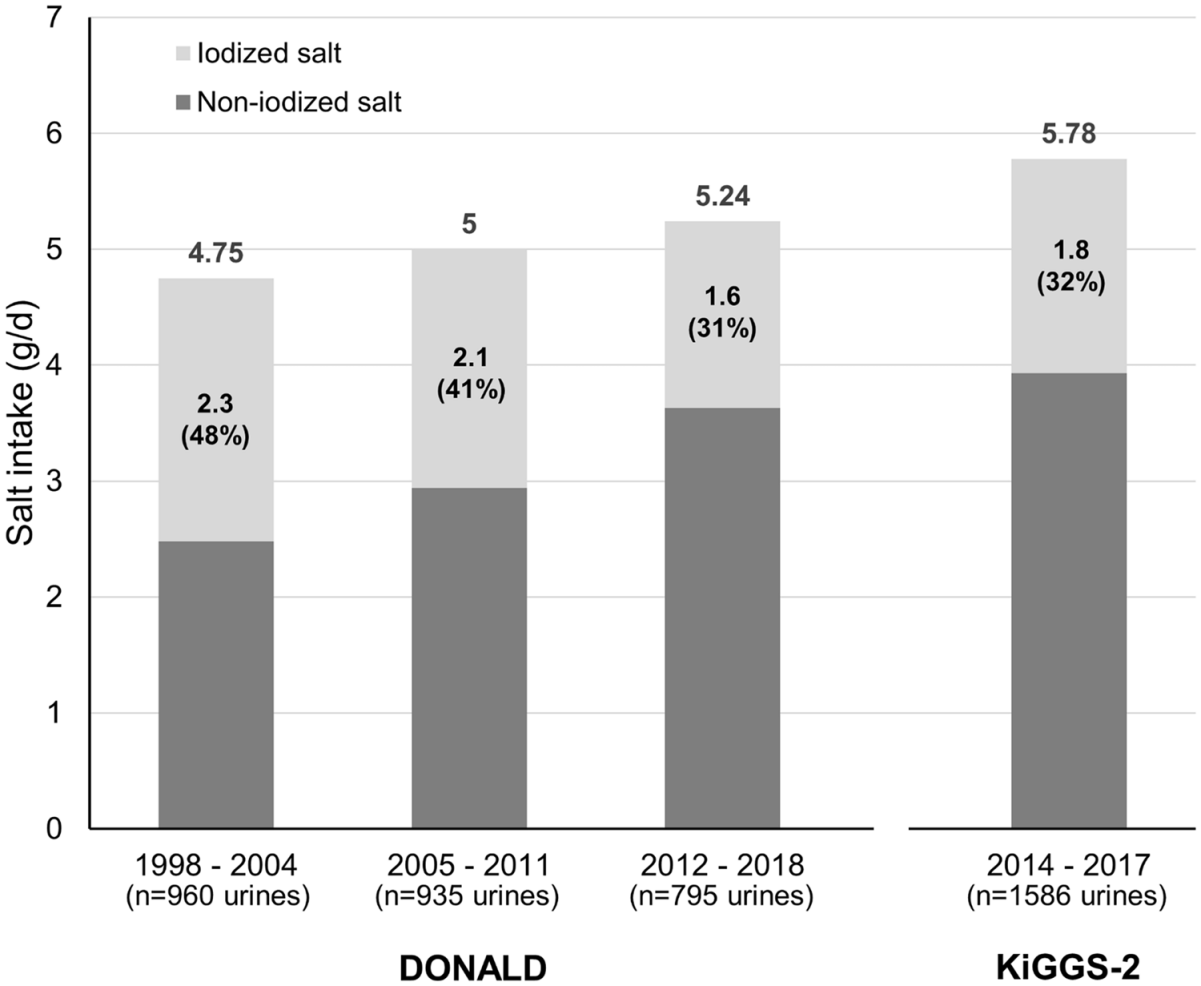
Changes in contribution of iodized salt to total salt intake in DONALD 6–12-year-old schoolchildren over 2 decades till 2018 and comparison with respective nationwide KiGGS-2 data. Estimation of the proportion of iodized salt intake to total salt intake was done by calculation of the median amount of urinary iodine stemming from total salt intake (i.e., sodium excretion) using regressions of predicted values according to Esche et al. [7] and Haldimann et al. [20] (see also Statistical analysis)

Figure 4 reveals that the increase in overall salt ingestion was paralleled by a clear reduction in iodized salt intake in DONALD children and that their attained percentage of iodized salt intake corresponds closely to the proportion determined in the latest German children’s survey.

## Discussion

In the last decades of the last century, only sporadic data were available on iodine status of German children. Several examinations on goiter prevalence and regional studies on iodine excretion in spot urine samples revealed mild to moderate iodine deficiency in Germany [21, 22]. However, systematic investigations in children were lacking up to the years 2003–2006 during which the first specific nationwide examination (KiGGS-1) covering the pediatric period was performed. Our detailed longitudinal analysis in the DONALD cohort, encompassing more than 30 years of systematic observation, did not only confirm the partly sufficient iodine status after 2003 as inferred from the KiGGS-1 data [23], but also revealed during which years in the past the corresponding improvement of iodine status began and when the recent impairment, as described in survey KiGGS-2, evolved.

The gold standard measurements of 24-h iodine excretion in closely followed children allowed us to substantiate the per-day iodine excretion results of the nationwide KiGGS surveys. In both KiGGS surveys 24-h urinary iodine excretion estimates were determined that incorporated individual creatinine excretion and individual body weight and allowed for sex- and age-stratified reference values for the muscularity marker creatinine [12]. Importantly, such advantageous estimates can be determined in each survey with simple spot urine collections, if creatinine concentration and body weight are additionally measured instead of solely determining spot iodine concentration.

In addition to the clear decrease in urinary iodine excretion of 6–8-year and 9–12-year-old children in KiGGS-2 with median levels falling below 60 µg/d and 75 µg/d, respectively, the DONALD cohort unveiled a significant increase in salt intake over the last 2 decades. This increase almost reached the average level of daily salt ingestion found in the nationwide survey. Using the creatinine related per-day excretion estimates it was also possible to calculate the approximate percentage of iodized salt to total salt intake. Again, the true per-day excretion measurements in the DONALD study with a derived estimate of 31 percent for the current proportion of iodized salt to total salt intake did validate the respective nationwide calculation from spot samples yielding 32%. In accord with the fact that long-term observations like DONALD allow to appraise the development of changes, e.g., of nutrient intake or food consumption over years or decades, the current analysis of the DONALD data reveals a considerable fall in the average use of iodized salt from almost 50% around the year 2000 to only ca. 30% nearly 15 years later (Fig. 4).

Long-term cohort studies can also provide in-depth information on effects and importance of particular legislative or administrative measures or population behavior changes for certain health- or nutrition-relevant outcomes. Accordingly, the efficacy of a legislative decree in 1993 in Germany is clearly discernible in the DONALD data (see Fig. 1). This decree enabled the utilization of iodized salt in non-industrially (e.g., bakeries and butcheries) and industrially produced foods without the clear restraint that had been effective before 1993. The fall of iodine excretion starting around 2013, as observable in the DONALD cohort too (Fig. 1), paralleled the trend of food manufacturers and distributors to reduce the use of iodized salt in convenience foods and major staples such as bread.

Several reasons may have led to an increasing reluctance of the food industry to use iodized salt in food production. Among them are (i) an increasing cost pressure and the higher price of iodized salt, (ii) trade barriers due to legislation variations in European countries, with countries not allowing iodized salt in processed foods or only permitting it in certain foods [24], (iii) an increasing propensity of both food industry and consumers to only have the pure ingredients and no additives in seasonings and foods (growing bio trend), (iv) a misconception that iodine is dangerous, e.g., as part of radioactive fallout in case of malfunctioning nuclear power plants, and (v) and probably also a decline in the awareness of iodine’s nutritional, health and cognitive importance [24]. Besides, the consumption of organically produced milk and dairy products which themselves contain measurably less iodine [25, 26] has steadily increased during the last 5–10 years.

A limitation of the present investigation is to be seen in the fact that 24-h urine collections were only available for the DONALD cohort. However, it has been shown that by referring analyte measurements in spot urine samples to additionally analyzed (individual) creatinine concentrations as well as to population-specific 24-h creatinine reference data, estimates of population medians can be attained that are closely comparable with the medians of true 24-h excretion rates for various analytes [12, 18, 23, 27–29]. This allows a reasonable comparability between real and estimated 24-h excretions.

Due to the rather elaborate study design involving periodic visits in the study center, the DONALD participants’ socioeconomic status is above average so that the full spectrum of dietary preferences may not be represented. While this contributes to some degree of selection bias, it does, on the other hand, clearly reduce the likelihood of residual confounding.

In conclusion, the present investigation illustrates the usefulness of non-representative longitudinal cohort studies collecting detailed biomarker-based prospective data to serve as sensitive sensors for health-relevant nutritional changes and trends in respective representative populations. Correspondingly, a current German downward trend in iodine supply was clearly detectable in advance of the completion of the nationwide survey KiGGS-2. In addition, the decrease in the use of iodized salt over more than 2 decades along with the steady increase in salt intake as determined biomarker-based in 24-h urine samples did substantiate the cross-sectional analyses in spot samples of the representative KiGGS study. Overall, using two different types of studies, a clear worsening of iodine nutrition in German children has been confirmed which is an alarming and unacceptable development.

Additionally worth mentioning is that on the one hand long-term cohort studies (that collect detailed physical, dietary, and biomarker data in children) allow to conduct unique physiological research on the interrelation of nutrition, metabolism, and growth [30–34], which in healthy children cannot be done in randomized controlled trials. And on the other hand such long-term cohort studies are valuable tools to identify, confirm, and substantiate changes and specific trends of different health-relevant outcomes like that of iodine status. Insufficient iodine status bears numerous disease risks such as hypothyroidism, goiter, thyroid nodules, and cognitive and developmental delay in children [35]. Hence, political efforts have to be urgently intensified that effectively re-strengthen awareness of both food industry and consumers for the importance of an improved and adequate iodine supply. Required is not only a clearly increased use of iodized salt for processed food production, but also an increase in iodine fortification of iodized salt, which at present is 20% lower in Germany (mean: 20 µg/g salt) than in Switzerland (mean: 25 µg/g salt) although in Switzerland the overall use of iodized salt is markedly higher [7].

## Supplementary Information

Below is the link to the electronic supplementary material.

Supplementary file1 (DOCX 13 KB)

## Data Availability

Although local data governing bodies at University of Bonn do not authorise individual level data from the DONALD cohort to be shared, the de-identified dataset supporting the conclusions of this article can be made available from the corresponding author upon reasonable request.

## Code availability

Computer code or algorithm (SAS commands in version 9.2) used to generate DONALD study results are available from the corresponding author upon request.
