# Supplementary material for: The DONALD study as a longitudinal sensor of nutritional developments: iodine and salt intake over more than 30 years in German children
Source: Eur J Nutr. 2022 Jan 18;61(4):2143–51. doi: 10.1007/s00394-022-02801-6 (PMC9106614; doi:10.1007/s00394-022-02801-6)
Supplement: Supplementary file 1 — Supplementary file1 (DOCX 13 KB) [file 394_2022_2801_MOESM1_ESM.docx]

| **Supplemental Table 1** Association of 24-h urinary iodine excretion with time, urine volume, and creatinine excretion in 6-12 years old participants of the DONALD study (Linear mixed-effects regression models (PROC MIXED)) | | | | | | | |
| --- | --- | --- | --- | --- | --- | --- | --- |
|  | Year periods | | | | | | |
|  | 1985-1992^a,b,c^ |  | 1993-2003^b,c^ |  | 2004-2012^b,c^ |  | 2013-2018^a,b^ |
| Year | 0.005 (–0.004, 0.015) |  | 3.51 (2.98, 4.03)^***^ |  | –0.58 (–1.32, 0.15) |  | –0.022 (–0.029, –0.015)^**^ |
| urine volume | 0.0001 (0.000013, 0.00019)^*^ |  | 0.023 (0.02, 0.03)^***^ |  | 0.026 (0.021, 0.031)^***^ |  | 0.00015 (0.00012, 0.00019)^***^ |
| creatinine | 1.70 (1.03, 2.36)^***^ |  | 188.59 (133.71, 243.47)^***^ |  | 239.29 (172.07, 306.50)^***^ |  | 1.39 (0.93, 1.85)^**^ |
| ^*^P < 0.05; ^**^P < 0.001; ^***^P < 0.0001. | | | | | | | |
| ^a^Outcome 24-h urinary iodine excretion was logrithmic transformed (log_10_ formation). | | | | | | | |
| ^b^Age was included in the regression model as a covariate and showed significant association with the outcome (P ≤ 0.02). | | | | | | | |
| ^c^Sex was included in the regression model as a dummy variable and showed significant association with the outcome (P ≤ 0.02) with boys having higher 24-h urinary iodine excretion rates than girls. | | | | | | | |
